# Response: “Commentary: Duration-dependent effects of the BDNF Val66Met polymorphism on anodal tDCS induced motor cortex plasticity in older adults: a group and individual perspective”

**DOI:** 10.3389/fnagi.2016.00028

**Published:** 2016-02-19

**Authors:** Rohan Puri, Mark R. Hinder

**Affiliations:** Sensorimotor Neuroscience and Ageing Laboratory, Faculty of Health, School of Medicine, University of TasmaniaHobart, TAS, Australia

**Keywords:** *BDNF*, Val66Met polymorphism, transcranial magnetic stimulation (TMS), transcranial direct current stimulation (tDCS), motor cortex, older adults, corticospinal excitability, plasticity

In a commentary on our recent paper (Puri et al., 2015), Shpektor et al. (2015) provide alternative interpretations on the effects of the *BDNF* Val66Met polymorphism on transcranial direct current stimulation (tDCS)-induced plasticity in older adults. Here we respond to several key issues raised in regard to our findings, and discuss broader implications for the field of non-invasive brain stimulation (NBS).

Shpektor et al. (2015) suggest that our findings, whereby older Met carriers (homozygous or heterozygous for the Met allele) exhibit a greater plastic response to 20 min of anodal tDCS compared to Val/Val homozygotes, may be seen as “controversial” as we reported an influence of the *BDNF* Val66Met polymorphism on plasticity at rest (i.e., not during task performance). However, a broader search of the extant literature suggests that the effects of the *BDNF* Val66Met polymorphism on tDCS-induced plasticity are varied, even before one considers the interaction of tDCS effects with neural activation resulting from performing a concurrent motor task (e.g., Fritsch et al., 2010). Specifically, Antal et al. (2010) reported a significant Genotype x Time interaction where greater motor evoked potential (MEP) amplitude was observed in Met allele carriers compared to Val/Val homozygotes at 25 and 60 min following anodal tDCS. Furthermore, Teo et al. (2014) reported a significant facilitation in MEPs post anodal stimulation only for Met carriers and not Val/Val homozygotes. Recently, Strube et al. (2015) reported a greater increase in MEPs for healthy Met carriers than Val/Val homozygotes after anodal tDCS (although this did not quite reach the *a-priori* level of statistical significance, *p* = 0.072, it was associated with a large effect size, *d* = 0.799). On the other hand, some studies do not report statistically significant differences between Met carriers and Val/Val homozygotes (Cheeran et al., 2008; Di Lazzaro et al., 2012; Fujiyama et al., 2014). However, as we alluded to in our paper, an absence of statistically significant effects should be interpreted with caution, especially when sample sizes are small and studies are not primarily designed to investigate *BDNF* polymorphism effects (e.g., Fujiyama et al., 2014).

In addition, there appears to be some preliminary evidence to suggest that the specific polarity of tDCS may interact with BDNF to elicit different magnitude changes in plasticity. That is, putative LTD-like effects induced by cathodal tDCS appear to be somewhat less affected by the *BDNF* Val66Met polymorphism (Cheeran et al., 2008; Antal et al., 2010; Di Lazzaro et al., 2012; Strube et al., 2015) than purported LTP-like effects induced by way of anodal tDCS (Antal et al., 2010; Teo et al., 2014; Puri et al., 2015; Strube et al., 2015). Large sample studies which directly assess the effect of tDCS polarity on BDNF-mediated plasticity effects would help elucidate the extent of this interaction. Overall, it must be concluded that the role of *BDNF* Val66Met polymorphism on mediating tDCS-effects remains unclear, and, that further studies with significantly greater power (sample sizes) are needed to further elucidate a number of factors that may contribute to the divergent results across the existing literature.

Another important consideration is the role that altered brain states and processes may play in mediating plastic responses. Indeed, it has been suggested that in stroke patients, rather than being detrimental, the Met allele interferes with maladaptive brain plasticity such that Val/Val and Met carrier stroke patients may not differ in their absolute ability for recovery (Di Lazzaro et al., 2015, Di Pino et al., 2016). It is conceivable that changes that occur as a result of the natural (healthy) aging process could, at least in part, explain the “novel” results we recently reported. Despite the potential of tDCS (and NBS in general) to slow the undesired effects of aging, such as cognitive decline and degradation of fine motor skills, very few studies have specifically examined older cohorts. Given the scarcity of research considering *BDNF* Val66Met polymorphism effects on NBS-induced plasticity in older populations, it is worth reviewing extant research that has investigated the effects of this polymorphism on cognitive and motor function in young and aged populations.

In young adults, the Met allele is often associated with *impaired* cognitive functioning, particularly in the memory domain (for meta-analysis see Kambeitz et al., 2012). In contrast, the Met allele appears to be less detrimental in older adults, and may even protect against the deleterious effects of aging to some degree. Specifically, older Met carriers demonstrate *improved* performance compared to Val/Val homozygotes on an array of cognitive tasks (Harris et al., 2006; Gajewski et al., 2011, 2012)^1^. Moreover, a 10 year longitudinal study in healthy older adults reported that Val/Val homozygotes exhibited significant *decline* in task-switching over the 10 year period whereas Met carriers' performance remained unchanged (Erickson et al., 2008).

Comparable age-effects are observed in the motor domain: Fritsch et al. (2010) and McHughen et al. (2010) report degraded motor performance in young Met carriers, where-as no detrimental effect of the Met allele was observed in motor behavior, neurophysiology, or use-dependent plasticity mechanisms for older adults (McHughen and Cramer, 2013). Overall, these studies highlight that the effects of the *BDNF* polymorphism are dynamic in nature and change during the normal aging process. Moreover, these findings suggest that the positive effect of the Met allele on the extent of tDCS-induced plasticity in our study may be partly reflected by the aged status of our cohort.

Our paper, with its limitations acknowledged therein, is one of the largest sample sized studies aimed to provide preliminary insights into the effects of this polymorphism in an older population. In light of aging demographics it therefore assumes considerable importance. However, further systematic investigations are needed to develop a clearer and deeper understanding while also providing replication. First and foremost, future studies should employ larger participant numbers and – even though most research has employed unbalanced samples – seek equal sample sizes of all three genotypic distributions (Val/Val, Val/Met, Met/Met). Finally, in addition to probing the genetic effects on artificially induced-plasticity using NBS and use-dependent plasticity using motor/cognitive training paradigms, understanding how the interaction between these two forms of plasticity is mediated by genotype would provide a novel basis to extend research on the effects of this polymorphism.

## Author contributions

RP and MRH prepared, wrote, and revised the manuscript.

## Funding

This research was supported under the Australian Research Council's Discovery Projects funding scheme (DP130104317) and by a DECRA fellowship (DE120100729) awarded to MRH.

### Conflict of interest statement

The authors declare that the research was conducted in the absence of any commercial or financial relationships that could be construed as a potential conflict of interest.

## Footnotes

^1^See Papenberg et al. (2015) for counter-argument.
